# GSK-3β coordinates axonal microtubule organization through Shot and Tau

**DOI:** 10.1073/pnas.2516746123

**Published:** 2026-02-17

**Authors:** André Voelzmann, Lubna Nuhu-Soso, Alex E. Roof, Sanjai Patel, Hayley Bennett, Antony Adamson, Gareth J. O. Evans, Marvin Bentley, Ines Hahn

**Affiliations:** ^a^School of Environmental and Life Sciences, Faculty of Science and Engineering, University of Hull, Hull HU6 7RX, United Kingdom; ^b^Manchester Academic Health Science Centre, Faculty of Biology, Medicine and Health, Division of Molecular and Cellular Function, School of Biological Sciences, University of Manchester, Manchester M13 9PL, United Kingdom; ^c^York Biomedical Research Institute and Department of Biology, University of York, York YO10 5DD, United Kingdom; ^d^Manchester Fly Facility, Faculty of Biology, Medicine and Health, School of Biological Sciences, University of Manchester, Manchester M13 9PL, United Kingdom; ^e^Genome Editing unit, Faculty of Biology, Medicine and Health, School of Biological Sciences, University of Manchester, Manchester M13 9PL, United Kingdom; ^f^Department of Biological Sciences and the Center for Biotechnology and Interdisciplinary Studies, Rensselaer Polytechnic Institute, Troy, NY 12180

**Keywords:** GSK-3β, microtubules, axons, neurodegeneration, neurodevelopment

## Abstract

Glycogen Synthase Kinase 3β (GSK-3β) is a key coordinator of neuronal development and maintenance; hyperactive GSK-3β is linked to neurodevelopmental and -degenerative diseases and therefore a promising therapeutic target. In neurons, GSK-3β coordinates the cytoskeleton by phosphorylating microtubule-binding proteins. In this study, we found that tight regulation of GSK-3β kinase activity is required for the maintenance of parallel microtubule bundles in *Drosophila* and rat axons. Up- or downregulation of GSK-3β led to axons forming pathological swellings in which microtubule bundles disintegrated into disorganized, curled microtubules. We identified the microtubule bundling proteins Shot and Tau as key GSK-3β targets and found that GSK-3β exerted its regulatory effect on microtubule bundling through them. GSK-3β regulates the ability of Shot and Tau to attach to microtubules and/or Eb1. Misregulation of GSK-3β leads to the loss of Eb1–Shot-mediated guidance of polymerizing microtubules into parallel bundles, thus causing disorganization. We propose that microtubule disorganization during both active and inactive states of GSK-3β links its hyperactivity to neurodegeneration and may explain why global GSK-3β inhibition has failed in clinical trials.

## Significance

Glycogen Synthase Kinase 3β (GSK-3β) is a crucial regulator of neuronal development and implicated in various neurodegenerative and neurodevelopmental diseases. Yet the cellular mechanisms linking its activity to neuronal development and maintenance remain unclear. We found that GSK-3β maintains axonal microtubule bundles by modulating the dual roles of the microtubule regulators Shot and Tau. Disruption of this balance, either through kinase overactivation or inhibition, leads to pathological unbundling of microtubules, impairing axonal integrity. This work reveals a fundamental role for GSK-3β in organizing the neuronal cytoskeleton, with implications across the diverse cell types and contexts in which GSK-3β functions. Our findings might help explain why broad GSK-3β inhibition has shown limited success in therapeutic settings.

The development and maintenance of axons critically depend on parallel microtubule bundles. Microtubules establish axonal projections, adapt neuronal cell shape, and are the substrate for motor proteins that move vesicles and other cargoes (1–3). The regulation of microtubule polymerization and stability is crucial for the formation of microtubule bundles, and disruptions to this process, such as microtubule loss or disorganization, are common features in many axon pathologies (3, 4). Despite their physiological importance, the molecular mechanisms that maintain microtubule bundles are not fully understood (5).

The kinase GSK-3β has emerged as a key regulator of microtubule stability and dynamics (6). GSK-3β is required for proper neuronal development, and misregulation of GSK-3β is linked to several neurodevelopmental and -degenerative disorders (7–11). GSK-3β is therefore a promising therapeutic target for neurological disorders. However, global GSK-3β inhibition has either not been therapeutically beneficial (12) or leads to long-term toxicity [e.g. approved inhibitor Lithium (13)] and neuronal decay (14), indicating a not yet appreciated complexity of GSK-3β regulation.

Here, we tested if misregulation of GSK-3β affects axonal microtubule bundles in primary neuron cultures from genetically tractable *Drosophila* (5, 15–19) and primary rat hippocampal neurons. We found that misregulation of GSK-3β, both hyper- and inactivation, disrupted the organization of parallel microtubule bundles. We identified two key microtubule-associated proteins, the spectraplakin Short Stop (Shot) and Tau, as essential GSK-3β targets for proper microtubule bundling. Both proteins play an important role in maintaining microtubule bundles (16, 18).

We found that hyperactivation of GSK-3β causes microtubule unbundling by reducing binding of Tau to microtubules, which disrupts the Shot/Eb1-mediated alignment of polymerizing microtubules into parallel bundles (18). In contrast, GSK-3β inactivation detaches the microtubule-actin crosslinker Shot from plus ends. This prevents Shot from guiding polymerizing microtubules into proper bundles. These differential effects of GSK-3β-mediated modulation of key microtubule regulators and their impact on microtubule bundle maintenance is a model to explain how hyper- and hypophosphorylation by GSK-3β causes pathology. Furthermore, this framework explains why global GSK-3β inhibition has yielded little to no therapeutic effect on neurodegenerative diseases.

## Results

### Activation and Inactivation of dGSK-3β Cause Disorganization of Axonal Microtubules in *Drosophila* Primary Neurons.

**Fig. 1.**
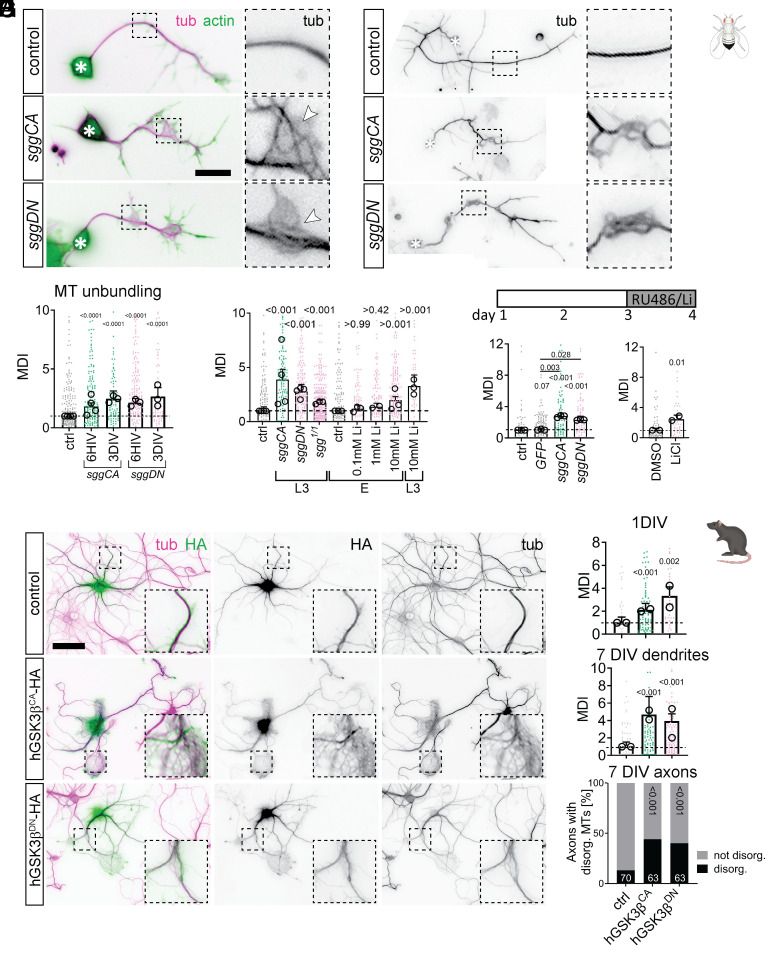
Inhibition and hyperactivation of GSK-3β/Shaggy both lead to curling and unbundling of axonal microtubules in *Drosophila* primary and rat hippocampal neurons. (*A* and *B*) Images of representative examples of *Drosophila* embryonic primary neurons either immuno-stained for tubulin and actin (*A*) or tubulin (*B*). Neurons of the following conditions were cultured for 6 h in vitro (6HIV, *A*) or 3 d in vitro (3DIV, *B*): controls (ctrl; *elavGal4*, *UAS-GFP*), expressing constitutively active (*sggCA*; *elavGal4, UAS-sgg^S9A^*) or inactive (*sggDN*; *elavGal4, UAS-sgg^A81T^*) *UAS-dGSK-3β* variants via the pan-neuronal driver *elavGal4*. Asterisks indicate cell bodies, dashed squares in are shown as 3.5-fold magnified close-ups beside each image white arrow heads point at areas of microtubule curling. (*C*) Quantification of microtubule unbundling depicted as MDI obtained from embryonic primary neurons with the same genotypes as shown in (*A, B*, and *D*) Microtubule unbundling quantification of primary neurons obtained from third instar larval brains (L3) expressing in/active dGSK-3β (*sggCA/DN*), embryonic primary neurons of dGSK-3β mutants (*sgg^1/1^*) and neurons treated with the GSK-3β inhibitor Lithium. (*E*) **Green Fluorescent Protein (*GFP*)**, *sggDN* or *sggCA* expression was induced via RU486 (elav::switchGal4) at three DIV for one DIV or control neurons were treated with 10 mM Li. (*F*) Images of representative examples of rat hippocampal neurons at seven DIV expressing HA (controls), hGSK-3β^CA^-HA or hGSK-3β^DN^-HA immuno-stained for tubulin (magenta) and HA (green). (*G*–*I*) Quantification of microtubule unbundling depicted as MDI (*G* and *H*) or ratio (axons w/ or w/o microtubule unbundling, *I*) at conditions indicated above. Data were normalized to parallel controls (dashed horizontal line) and are shown as mean ± SEM; data points in each plot, taken from at least two experimental repeats consisting of three replicates each; large open circles in graphs indicate median/mean of independent biological repeats. *P*-values obtained with Kruskall-Wallis ANOVA test for the different genotypes are indicated in each graph. Scale bar in (*A*) represents 10 μm in (*A*) and 20 μm in (*F*). For raw data, see Dataset S1.

To evaluate a role of GSK-3β in axonal microtubule organization, we expressed constitutively active or dominant-negative *Drosophila* GSK-3β (dGSK-3β or Shaggy) mutants in cultured neurons and analyzed microtubule organization. Primary neurons were derived from embryos of fly lines overexpressing either constitutively active dGSK-3β [CA, *UAS-sggCA*, S9A mutation (20)] or dominant-negative, kinase-dead [*UAS-sggDN*; A81T mutation (20)]. After 6 h in vitro (HIV), neurons were fixed and immuno-stained for tubulin to visualize microtubules. Activation or inhibition of dGSK-3β caused axon swellings where microtubules lost their bundled conformation and became curly (Fig. 1*A*). We quantified this phenotype using the previously described microtubule disorganization index (MDI; area of microtubule disorganization relative to axon length; see refs. 17, 18, 21, 22). The MDI measures the fold-increase of microtubule unbundling relative to control neurons. The measurements resulted in an MDI of 1.8 for *sggCA* and 2.1 for *sggDN* (Fig. 1*C*). To independently verify the impact of dGSK-3β inactivation on microtubule organization, we cultured neurons from animals with a dGSK-3β loss-of-function allele [*sgg^1^*, (23)] or treated wild-type neurons with increasing concentrations of the GSK-3β inhibitor lithium [Li, (24)]. Both treatments resulted in a significant increase in microtubule unbundling (*sgg^1/1^* MDI = 1.8; 10 mM lithium MDI = 3.3) (Fig. 1*D*).

To determine if dGSK-3β’s role in microtubule organization extends beyond early growth cone stages, we analyzed neurons expressing *sggCA* or *sggDN* after three days in vitro (three DIV, Fig. 1*B*). Older neurons showed increased microtubule curling in both conditions (*sggCA* MDI = 2.5; *sggDN* MDI = 2.7) (Fig. 1*C*). Neurons cultured from larval brains also exhibited significant unbundling with *sggCA* (MDI = 3.9) and *sggDN* (MDI = 2.8) (Fig. 1*D*).

We next analyzed if altering dGSK-3β levels induces microtubule unbundling in mature neurons using RU486-inducible Gal4/UAS expression (*elavGS/UAS-sggCA* or -*sggDN*) (25). Neurons matured three DIV without induction to post growth cone stage where they form presynapses (26) before induction with 100 µg/mL RU486 for 24 h. Controls showed minimal disorganization, whereas sggCA (MDI = 2.7) and sggDN (MDI = 2.3) significantly increased microtubule unbundling (Fig. 1*E*). As an orthogonal approach, we treated three-day-ol, cultured neurons with the GSK-3β inhibitor Li for 24 h. 10 mM Li treatment resulted in a 2.3-fold increase of microtubule disorganization relative to vehicle-treated neurons Dimethyl Sulfoxide (DMSO) which is comparable to expression of constitutively active dGSK-3β (Fig. 1*E*).

Together, these results indicate that both in- and decrease of dGSK-3β kinase activity causes microtubule disorganization. Therefore, balanced GSK-3β kinase activity is required for maintenance of the microtubule network in growing and mature neurons.

### hGSK-3β-Mediated Regulation Is Required for Maintaining Microtubule Organization/Bundling in Mammalian Neurons.

To explore whether this observation applies universally, we determined if misregulation of human GSK-3β (hGSK-3β) affects microtubule organization in cultured rat hippocampal neurons. We electroporated neurons with constitutively active (*HA-GSK-3β^CA^*) or dominant-negative (*HA-GSK-3β^DN^*) hGSK-3β plasmids before plating (0 DIV). Neurons were fixed at one DIV and stained for α-tubulin and HA. At one DIV, most neurons are in developmental stage 2, having extended minor neurites but prior to polarization and the differentiation of axon and dendrites (27). Expression of constitutively active (MDI = 2.1) and dominant negative (MDI = 3.3) hGSK-3β resulted in a substantial increase in microtubule disorganization (Fig. 1 *F* and *G*).

To test whether hGSK-3β maintains microtubule organization in mature neurons, we transfected six DIV hippocampal neurons with constitutively active or dominant-negative GSK-3β and fixed 24 h later. At seven DIV, neurons are fully polarized with developed axons and dendrites (27). Both constructs markedly increased microtubule disorganization compared to controls (HA-GSK-3βCA MDI = 4.6; HA-GSK-3βDN MDI = 3.9; Fig. 1 *F* and *H*). Dendritic MDI rose 4.6- and 3.9-fold, respectively. We were not able to calculate the MDI for axons, because axons of seven DIV neurons extend substantially beyond the field of view and the entire axon length is a required MDI parameter. Instead, we calculated the percentage of axons with disorganized MTs within each field of view and observed an increase from 13% in untreated neurons to 44% (GSK-3β^CA^) and 40% (GSK-3β^DN^) (Fig. 1*I*).

These experiments show that the effects we saw after modifying dGSK-3β activity in *Drosophila* were recapitulated in mammalian neurons. Therefore, balanced GSK-3β activity is required to maintain microtubule bundles is a conserved feature and likely a universal mechanism for maintaining the neuronal cytoskeleton.

### dGSK-3β Affects Eb1 Comet Formation Differentially.

**Fig. 2.**
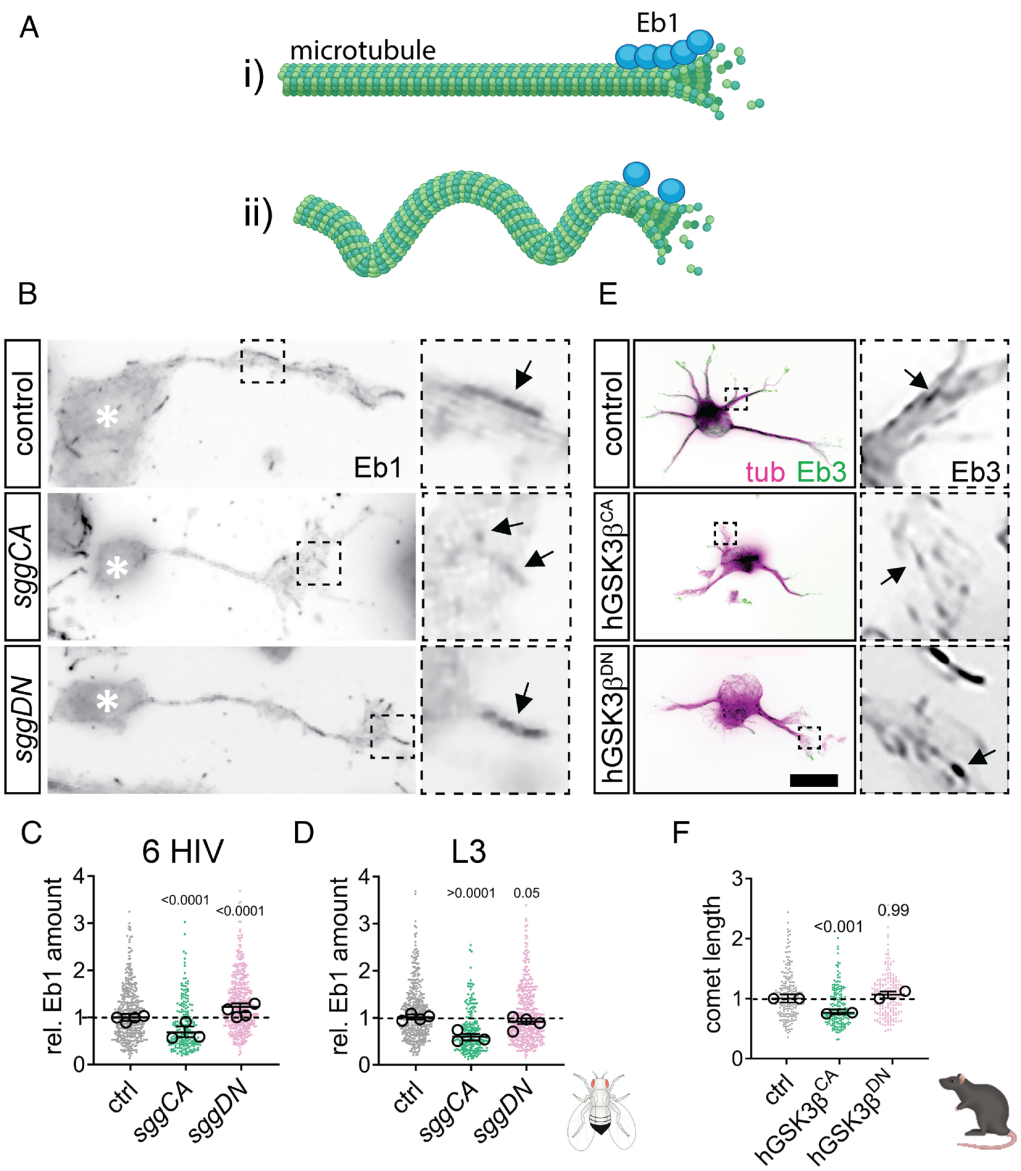
Expression of active GSK-3β reduces Eb1 comet size. (*A*) Schematic representation of how loss of Eb1 leads to microtubule unbundling (generated with BioRender): (*i*) normal Eb1 levels support microtubule polymerization and straight microtubule arrangement, whereas (*ii*) reduced Eb1 levels lead to microtubule curling. (*B*) Images of representative examples of control embryonic primary neurons or neurons expressing constitutively active (*sggCA*) or inactive dGSK-3β (*sggDN*) immuno-stained for Eb1. Asterisks indicate cell bodies, dashed squares in are shown as 3.5-fold magnified close-ups beside each image arrows point at Eb1 comets. Scale bar in *B* represents 10 μm. (*C* and *D*) Quantification of normalized Eb1 amounts at plus ends obtained from embryonic primary neurons at six HIV and larval neurons after one DIV (L3). (*E*) Representative images of primary rat hippocampal neurons at one DIV coexpressing Eb3-GFP with HA (controls), hGSK-3β^CA^-HA or hGSK-3β^DN^-HA. Asterisks indicate cell bodies, dashed squares in are shown as 3.5-fold magnified close-ups beside each image arrows point at Eb1 comets (*F*) Quantification of normalized Eb1 comet lengths obtained from the same genotypes as shown in (*E*). Data were normalized to parallel controls (dashed horizontal line) and are shown as mean ± SEM; data points in each plot, taken from at least two experimental repeats consisting of three replicates each; large open circles in graphs indicate median/mean of independent biological repeats. *P*-values obtained with Kruskall-Wallis ANOVA test for the different genotypes are indicated in each graph. Scale bar in *A* represents 10 μm in *B* and 20 μm in *F*. For raw data, see Dataset S2.

Eb1 is a master regulator for coordinating microtubule dynamics which binds to growing microtubules and acts as a scaffold that recruits other proteins (+TIPs) to microtubule tips. We previously found that microtubule bundle arrangement is tightly linked to the availability of Eb1 at microtubule plus ends, as there is a negative correlation between the size of Eb1 comets and microtubule curling: Smaller Eb1 comets result in larger MDI values (Fig. 2*A*) (18). We therefore tested if GSK-3β hyper- or inactivation depleted Eb1 at microtubule plus ends.

We cultured embryonic or larval neurons expressing constitutively active or dominant negative dGSK-3β and determined the amount of Eb1 at microtubule plus ends by immuno-staining (Fig. 2 *B*–*D*). Eb1 at plus ends decreased with sggCA in embryonic (six HIV, 67%) and larval (L3, 58%) neurons, while sggDN had only mild effects. Embryonic neurons showed higher Eb1 at tips, with no change in larval neurons. To assess whether hyperactive or inactive dGSK-3β influences *Eb1* expression, we compared *Eb1* mRNA levels in L3 wandering-stage brains expressing elav-driven *UAS-GFP*, *UAS-sggCA*, or *UAS-sggDN* using real-time RT-PCR. No significant differences in *Eb1* levels were observed among the conditions (*SI Appendix*, Fig. S1 *A* and *B*).

To test if GSK-3β manipulation had comparable effects in mammalian neurons, we coexpressed active hGSK-3β^CA^ or inactive hGSK-3β^DN^ with EB3-GFP, fixed and stained cells for GFP in one DIV neurons (Fig. 2 *E* and *F*). We did not observe a difference in comet length in neurons expressing hGSK-3β^DN^. However, hGSK-3β^CA^ causes reductions in comet size (75% of control).

These experiments suggest that loss of Eb1 from microtubule plus ends could explain the microtubule unbundling that is caused by GSK-3β^CA^ expression, but not GSK-3β^DN^. This indicates that the microtubule disorganization phenotypes induced by hyper- or inactivation of GSK-3β are caused by different mechanisms.

### dGSK-3β Hyperactivation Leads to Microtubule Disorganization Via Loss of Tau.

The microtubule-associated protein Tau is a GSK-3β phosphorylation target (28–30). Upon phosphorylation, Tau detaches from microtubules (31, 32) and loss of Tau from microtubules leads to decrease of Eb1 at plus ends resulting in microtubule unbundling (18). We hypothesized that GSK-3β hyperactivation causes microtubule disorganization by phosphorylating Tau and subsequent Tau detachment from microtubules.

**Fig. 3.**
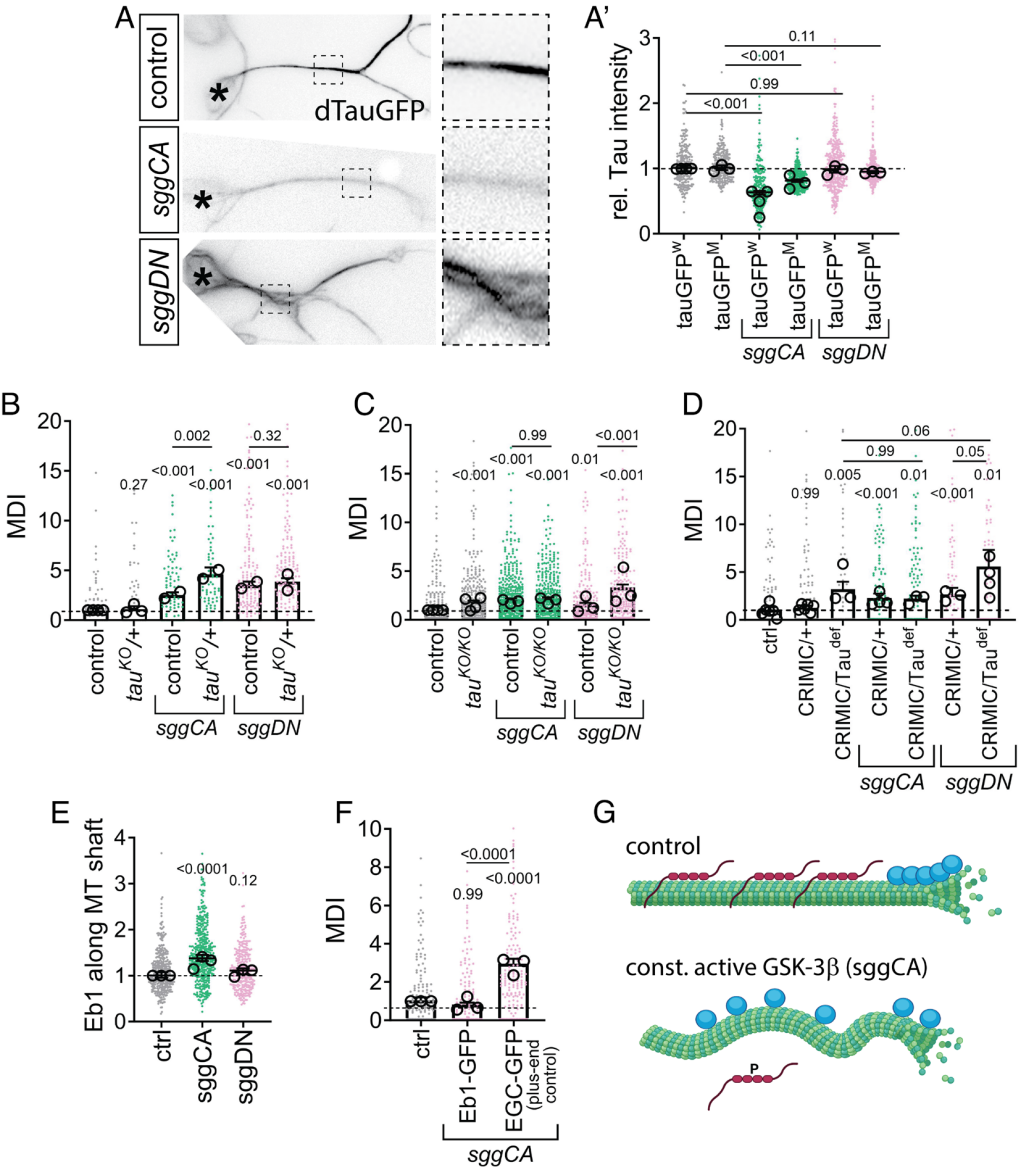
Reduced Eb1 at plus ends through a loss of Tau leads to microtubule unbundling when dGSK-3β is hyperactive. (*A*) Representative images of primary neurons expressing dTau-GFP [P{Wee}tau(304)] at endogenous levels, alone or in combination with active (*UAS-sggCA*) or inactive (*UAS-sggDN*) dGSK-3β expression. Asterisks indicate cell bodies, dashed squares are shown as 3.5-fold magnified close-ups beside each image. (*A’*) Quantification of relative Tau mean intensity along microtubules in two independent dTau-GFP lines, [P{Wee}tau(304)] (*tauGFP^W^*) and Mi{MIC}tauMI03440 (*tauGFP^M^*); data are normalized to control. (*B*–*D*) Microtubule curling for primary neurons displaying heterozygous (*B*) and homozygous (*C*) hypomorphic *tau^KO^* mutant or *tau^CRIMIC^*(CR70012)/*tau^Def^* [Df(3R)BSC49] (*D*) conditions, alone or in combination with expression of active (*UAS-sggCA*) or inactive (*UAS-sggDN*) dGSK-3β via *elavGal4* (*B* and *C*) or *tauCRIMIC*-induced Gal4 expression via *tau* gene regulatory elements (*D*). (*E*) Relative Eb1 intensity along microtubules of neurons at six HIV without/with *elav-Gal4*-driven expression of *sggCA* or *sggDN*. (*F*) Microtubule curling (MDI) in primary neurons expressing *sggCA* in combination with *UAS-Eb1-GFP* or -*EGC-GFP* (Shot C terminus). (*A’–E*) All data were normalized to parallel controls (dashed horizontal lines) and are shown as bar chart with mean ± SEM of at least two independent repeats with three technical replicates each; large open circles in graphs indicate mean of independent biological repeats. *P*-values above data points/bars were obtained with Kruskal–Wallis ANOVA tests. For raw data, see Dataset S3. (*G*) Model view of the results shown here; note that microtubules are depicted (green), blue circles represent Eb1 and red chains Tau. Scheme generated with Biorender.

To test whether GSK-3β activity affects Tau association with axonal microtubules, we expressed hyperactive sggCA or inactive sggDN in two independent fly lines where endogenous Tau is GFP-tagged (33, 34), cultured neurons, and performed live imaging. In controls, Tau-GFP localized to axonal microtubules (Fig. 3), but was reduced by 38% (Tau^W^-GFP) or 20% (Tau^M^-GFP) with sggCA expression, while sggDN had no effect. *Tau* mRNA levels were unchanged (*SI Appendix*, Fig. S1 *C* and *D*), indicating that dGSK-3β hyperactivation displaces Tau from axonal microtubules, supporting the hypothesis that GSK-3β overactivity disrupts microtubules via Tau misregulation. Together, this suggests that dGSK3b hyperactivation leads to loss of Tau from axonal microtubules and this finding is consistent with the hypothesis that GSK-3β overactivity causes microtubule disorganization through misregulation of Tau.

To test whether dGSK-3β and Tau act in the same pathway, we performed genetic interaction experiments using *tau* heterozygous flies (*tau^+/–^*; hypomorphic *tau^KO^* allele) (35) and measured microtubule disorganization (Fig. 3*B*). *tau^+/–^* alone had no effect, while *sggCA* increased disorganization (MDI = 2.5). This was strongly enhanced in *tau^+/–^* neurons (MDI = 4.6). *sggDN* also increased disorganization (MDI = 3.5), but this was not further affected by *tau* reduction (MDI = 3.9). These results support that *sggCA*, but not *sggDN*, genetically interacts with *tau*, indicating they may act in the same pathway.

To determine if dGSK-3β-mediated microtubule disorganization depended on Tau, we expressed *sggCA* in two independent *tau* loss-of-function fly lines and measured microtubule disorganization (Fig. 3 *C* and *D*). If *sggCA* acts through *tau*, its expression in homozygous *tau^–/–^* neurons should not increase disorganization. Indeed, *sggCA* expression had little effect in homozygous, hypomorphic *tau^KO^* (MDI = 1.9 and 2.0, respectively) or *tau^CRIMIC^/tau^Def^* mutants (near to complete loss of tau; MDI = 2.2 and 2.1). In contrast, *sggDN* expression in *tau^−/−^* neurons increased microtubule disorganization compared to *tau^−/−^* neurons from MDI = 1.4 to 3.1 in hypomorphic *tau^KO^* mutants and from MDI = 2.6 to 5.5 in *tau^CRIMIC^/tau^Def^* mutants (Fig. 3 *C* and *D*). These results support that dGSK-3β hyperactivation disrupts microtubules through Tau, whereas inactivation acts via a different mechanism.

We previously demonstrated that loss of Tau results in increased Eb1 binding to the microtubule lattice and loss of Eb1 at growing microtubule tips. Loss of Eb1 at plus ends leads to microtubule curling, because it reduces the ability of the actin-microtubule crosslinker Shot to guide growing microtubules into parallel bundles (18). To determine if hyperactive dGSK-3β leads to microtubule unbundling by Eb1 relocating from microtubule tips to the shaft, we stained for Eb1 in wt, *sggCA*-, or *sggDN*-expressing neurons, and measured Eb1 at microtubule shafts (Fig. 3*E*). We found that expression of *sggCA* resulted in a 1.3-fold increase of Eb1 at microtubule shafts (areas without comets) compared to control neurons (Fig. 3*E*). Expression of *sggDN* did not change Eb1 levels at microtubule shafts.

In *tau^−/−^* mutants, increasing Eb1 levels by overexpressing Eb1-GFP can restore Eb1 to the plus tip and rescue microtubule curling (18). Therefore, we asked if *Eb1-GFP* overexpression could rescue the microtubule disorganization phenotype caused by *sggCA* (Fig. 3*F*). Expression of *sggCA* did not cause microtubule disorganization (MDI = 0.8) when *Eb1-GFP* was coexpressed. This rescue is specific for Eb1, because coexpression of the plus-end binding domain of another protein [Shot, EGC (16)] and *sggCA* still resulted in microtubule disorganization (MDI = 2.92). This shows that dGSK-3β hyperactivation causes microtubule disorganization through Tau and Eb1 (Fig. 3*G*).

### Shot and Inactive dGSK-3β Act in the Same Pathway to Cause Microtubule Unbundling.

A key mediator of microtubule bundling is the Spectraplakin Short Stop (Shot) which guides polymerizing microtubules into parallel bundles by crosslinking cortical actin and Eb1(16). Therefore, Shot is a plausible target for dGSK-3β misregulation to cause microtubule disorganization.

**Fig. 4.**
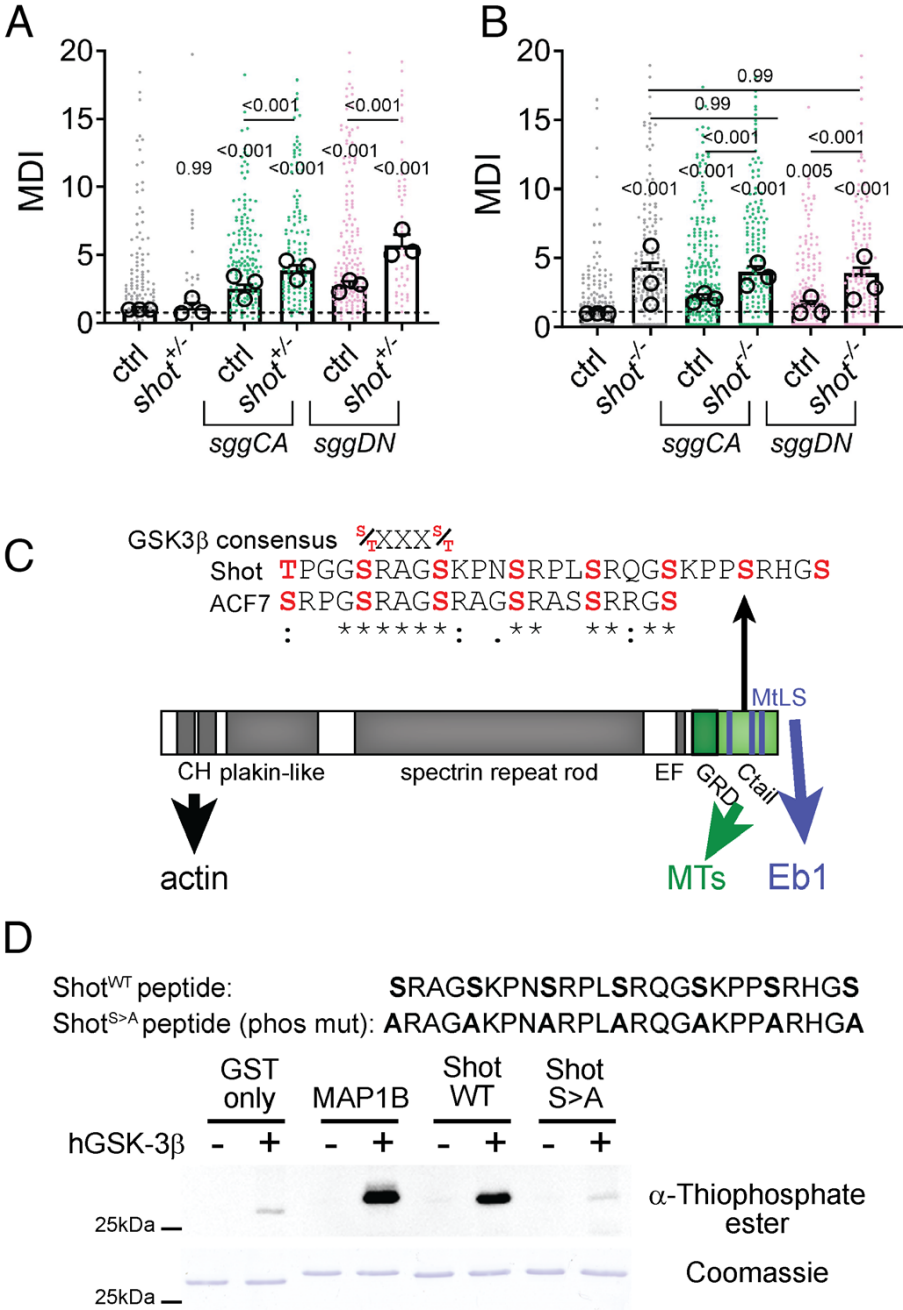
The C-terminus of Shot can be phosphorylated by GSK-3β. (*A* and *B*) Microtubule curling for primary neurons displaying heterozygous (*A*) and homozygous (*B*) *shot*^3^ mutant conditions, alone or in combination with expression of active (*UAS-sggCA*) or inactive (*UAS-sggDN*) GSK-3β. (*C*) Schematic representation of Shot; highlighting the verified ACF7 and putative Shot GSK-3β target site (consensus S/T S/T) in the C-terminal microtubule binding region. (*D*) In vitro thiophosphorylation kinase assay and Shot^WT^ and phosphodeficient Shot^S > A^ peptide sequence. Representative blot where phosphorylated peptides are labelled with α-Thiophosphate ester of GST control, positive control [GSK-3β target site of MAP1B, ERLSPAKSPSLSPSPPSPIEKT (37)], Shot^WT^ and Shot^S > A^ with or without hGSK-3β. Quantification of mean band intensity and full blots see *SI Appendix*, Fig. S2. For raw data see Dataset S4.

To test whether *dGSK-3β* and *shot* act in the same pathway, we reduced *shot* expression (*shot^+/–^*) and measured microtubule disorganization (Fig. 4*A*). *shot^+/–^* neurons showed no difference from the wild type (WT), indicating reduced *shot* is sufficient for bundling. However, expression of *sggCA* and *sggDN* increased disorganization to a greater degree in *shot^+/–^* neurons (MDI = 3.88 and 5.73) than in the WT (MDI = 2.53 and 2.75). To further assess sggDN’s dependence on Shot, we expressed *GSK-3β* in homozygous *shot^−/−^* neurons. *Shot* deletion alone caused severe disorganization (MDI = 4.14), and neither *sggCA* nor *sggDN* increased this further (MDI ≈ 3.8 to 3.7) (Fig. 4*B*). The observations that *shot* knockout alone achieved maximal disorganization and that disorganization was not enhanced by concurrent expression of *sggCA* or *sggDN*, suggest that GSK-3β manipulations that increase bundling are mechanistically linked to *shot* activity. These results are also strong evidence that Shot and dGSK-3β act in the same pathway.

### GSK-3β Phosphorylates Shot in its Eb1/Microtubule-Binding Region.

We found that sggCA or sggDN did not affect *shot* expression (*SI Appendix*, Fig. S1*E*) and therefore assessed if GSK-3β can regulate Shot directly. GSK-3β phosphorylates serine/threonine residues of defined consensus motifs (36). In the mammalian Shot homologue ACF7, GSK-3β phosphorylates a cluster of serines that follow this pattern (Fig. 4*C*). They are located in between the C-terminal microtubule binding domain (Gas domain) and Eb1 protein-binding SxI/LP sites. We identified a comparable cluster of putative GSK-3β phosphorylation sites in the C terminus of Shot by sequence analysis (residues 5,036 to 5,064 of Shot-PE, Fig. 4*C* and *SI Appendix*, S2*A*).

To determine if GSK-3β phosphorylates these residues, we generated a GST-tagged peptide of the WT cluster (Shot^WT^) and serine-to-alanine mutant (Shot^S > A^), along with a MAP1B peptide known to be phosphorylated by GSK-3β [ERLSPAKSPSLSPSPPSPIEKT; (37)] as a positive control. We tested these peptides in a GSK-3β thiophosphorylation assay (38) (Fig. 4*D*). We used hGSK-3β, because fly and mammalian GSK-3β show a high-level structural and functional conservation and the kinase and its targets have been used interchangeably (39, 40). We observed minimal GST phosphorylation, robust MAP1B phosphorylation, and substantial Shot^WT^ phosphorylation. Shot^S > A^ was not efficiently phosphorylated, indicating that the serine cluster is required (Fig. 4 and quantification in *SI Appendix*, S2*B*’). These data show that hGSK-3β can phosphorylate the C-terminal tail of Shot.

### Shot Phosphorylation Is Required to Maintain Axonal Microtubule Organization.

**Fig. 5.**
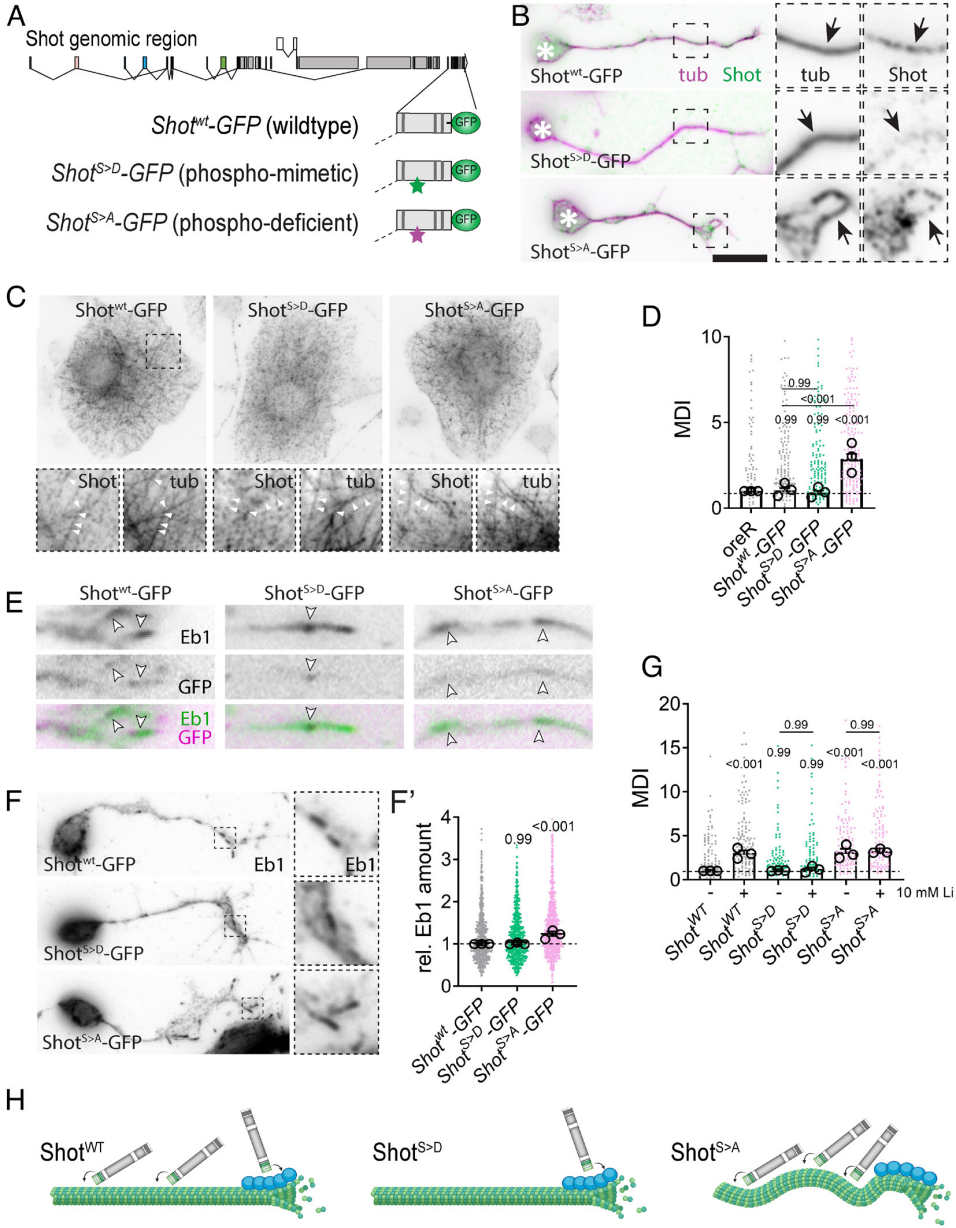
Phosphodeficient but not -mimetic Shot leads to microtubule unbundling. (*A*) Schematic representation of the genomic engineering approach replacing C-terminal exons of *Shot* to generate endogenous GFP-tagged Shot CRISPR lines, as either WT (*Shot^WT^*), phosphomimetic (*Shot ^S > D^*) or -deficient (*Shot^S > A^*) variants. (*B*) Representative images of *Drosophila* primary neurons and (*C*) non-neuronal, fibroblast-like cells cultured for six HIV of *Shot^WT^*, *Shot^S > D^* or *Shot ^S > A^* labeled for tubulin (purple) and Shot (green, anti-GFP). Asterisks indicate cell bodies, dashed squares are shown as 3.5-fold magnified close-ups, arrows/white arrowheads point at microtubules. (*D*) Quantification of microtubule unbundling (MDI) obtained from embryonic primary neurons with conditions indicated in *B*). (*E, F*) Drosophila primary neurons labelled for Eb1 (green) and GFP (magenta) in *E*) and black in *F*). White arrowheads point at Eb1 comets. (*F*’) Quantification of relative Eb1 amounts at plus ends for conditions indicated. *G*) Quantification of microtubule unbundling (MDI) obtained from primary neurons treated for the entire 6HIV with 10 μM Li or vehicle. All data were normalized to parallel controls (dashed horizontal lines) and are shown as bar chart with mean ± SEM of at least two independent repeats with three technical replicates each; large open circles in graphs indicate mean of independent biological repeats. P-values above data points/bars were obtained with Kruskal–Wallis ANOVA tests. (*H*) Cartoons, generated by Biorender, illustrating current hypothesis how phosphorylation by GSK-3β affects Shot’s ability to bind plus ends or microtubules. For raw data, see Dataset S5.

Because GSK-3β can phosphorylate Shot, we asked if Shot phosphorylation was important for axonal microtubule organization. We modified the *shot* locus and generated three different fly lines where the final three exons were replaced with a merged single exon that also encoded a C-terminal GFP (Fig. 5 *A* and *SI Appendix*, S3*A*): i) WT, ii) phosphomimetic (seven serines replaced with aspartic acids, Fig. 4 *C*), and iii) phospho-null (serines replaced with alanines). Normal expression of *shot* in those lines was confirmed via real-time RT-PCR (*SI Appendix*, Fig. S3*B*).

We cultured primary neurons from homozygous embryos of each line for six HIV and stained for microtubules and GFP to enhance the Shot-GFP signal (Fig. 5*B*). In all conditions, Shot-GFP was expressed at low levels and exhibited a punctate distribution in axons. Shot^WT^-GFP puncta frequently colocalized with microtubules, although the low intensity of Shot-GFP puncta prevented systematic colocalization quantification. In contrast, phosphomimetic Shot^S > D^-GFP exhibited little colocalization with microtubules. Phosphodeficient Shot^S > A^-GFP localized to microtubules, comparable to Shot^wt^-GFP (more examples in *SI Appendix*, Fig. S4). We performed complementary experiments in *Drosophila* fibroblast-like cells cocultured with the primary neuron population and found comparable results. Shot^WT^-GFP and Shot^S > A^-GFP colocalized with microtubules, but Shot^S > D^ did not (Fig. 5*C*). These results support the hypothesis that Shot’s ability to bind microtubules is regulated by GSK-3β-mediated Shot phosphorylation.

We next determined if the phosphorylation state of Shot affects microtubule bundling. We measured microtubule disorganization by MDI and found that neurons from homozygous *shot^WT^* and *shot^S > D^* embryos exhibited no substantial microtubule disorganization (Fig. 5*D*). In contrast, neurons cultured from phosphodeficient *shot^S^*^>^*^A^* mutants showed significant microtubule disorganization (MDI = 2.9).

Even though Shot^S > A^-GFP, in contrast to Shot^S > D^-GFP, binds microtubule efficiently, the phosphodeficient S > A mutation leads to microtubule disorganization. This shows that Shot binding of the microtubule shaft is not linked with microtubule disorganization. An explanation for microtubule unbundling in phospho-deficient Shot^S > A^-GFP neurons could therefore come from Shot’s interaction with Eb1. If Shot cannot bind Eb1, it loses its ability to guide polymerizing microtubules into parallel bundles (16). To test if Shot^S > A^-GFP leads to microtubule unbundling due to reduced Shot–Eb1 interaction, we stained the three lines for Eb1. We found that Shot^S > D^-GFP, but not Shot^S > A^-GFP, colocalized with Eb1, (Fig. 5*E*; low intensity of shot-GFP puncta prevented systematic colocalization quantification). These results support the hypothesis that Shot^S > A^-GFP cannot efficiently bind Eb1.

Previous work found that loss of Shot–Eb1 interaction causes increased Eb1 comet sizes because microtubule-bound Shot slows microtubule polymerization (16). To determine if the phosphorylation state of Shot affected Eb1 binding to microtubule plus ends, we quantified the comet size in each condition. Shot^S > D^-GFP expressing neurons did not show any effect on comet size (Fig. 5*F’*). In contrast, Eb1 comets were significantly larger in the presence of phosphodeficient Shot^S > A^-GFP. Taken together, these results support the hypothesis that Shot–Eb1 interaction is lost when Shot is not phosphorylated, which leads to a loss of Shot-mediated microtubule guidance and microtubule disorganization.

To determine if Shot and dGSK-3β act in the same pathway, we used our *shot-GFP* mutant fly lines and assessed microtubule unbundling with or without GSK-3β inhibition (10 mM LiCl). If effects were additive, they would act independently; if not, they share a mechanism. Lithium treatment increased unbundling threefold in Shot^WT^ neurons but had no effect in Shot^S > A^ (MDI = 3.14 vs 3.23) and did not induce disorganization in Shot^S > D^ (MDI = 1.04 vs 1.2) (Fig. 5 *G*). Thus, GSK-3β inhibition and loss of Shot phosphoregulation are not additive, indicating that dGSK-3β acts through Shot.

### Defects in Locomotor Behavior Through Activation and Inactivation of dGSK-3β is Linked to Microtubule Unbundling.

**Fig. 6.**
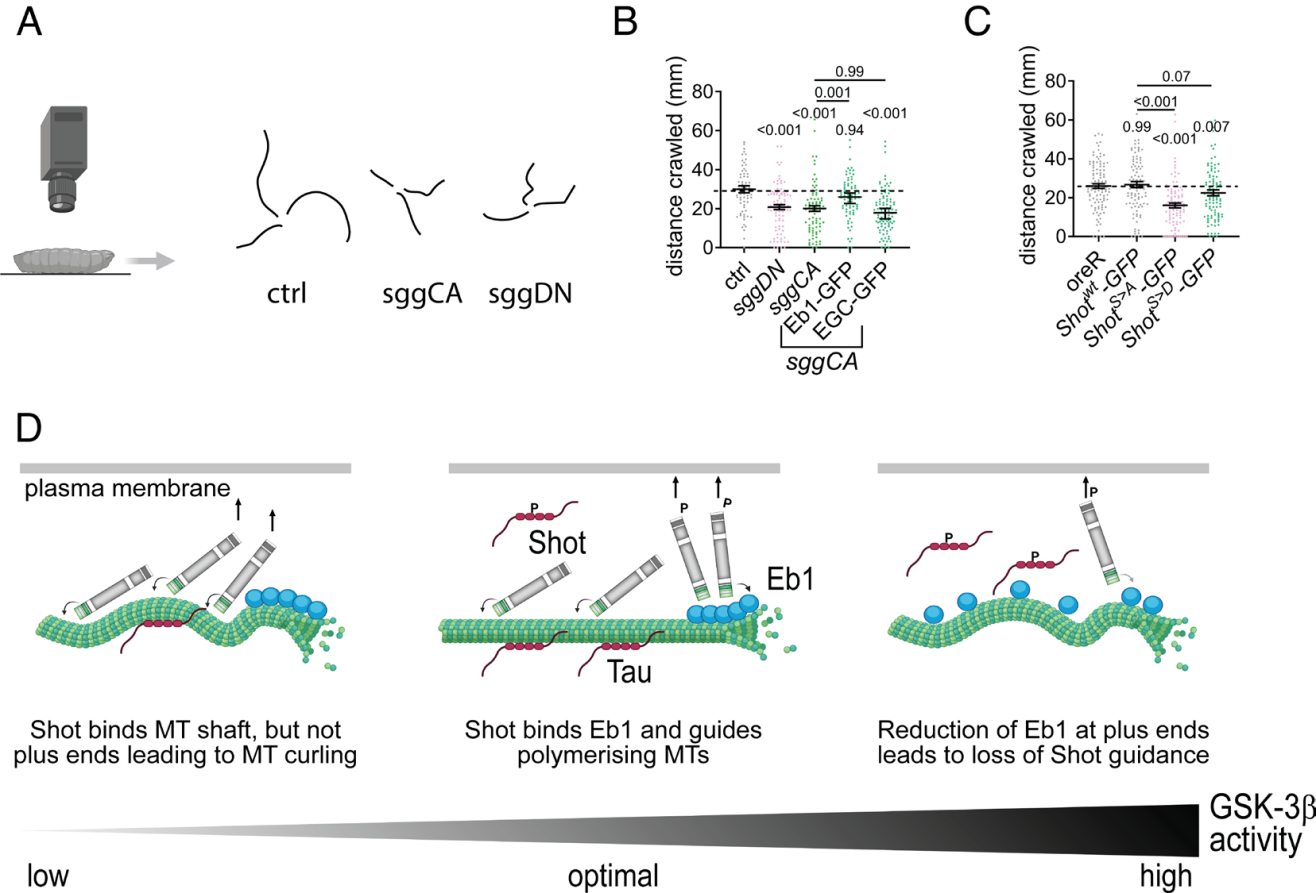
GSK-3β dysregulation impairs locomotion behavior via microtubule unbundling. (*A*) Schematic depicting larval crawling analysis and example crawling tracks recorded over a 60-s period from lines expressing *GFP* (control), *sggCA* or *sggDN*. (*B* and *C*) Summary data (median ± SEM) quantifying distance crawled. Each point represents the distance traveled by an individual animal. *P*-values above data points were obtained with Kruskal–Wallis ANOVA tests. For raw data, see Dataset S6. (*D*) GSK-3β activity needs to be tightly regulated to control microtubule bundling—a mechanistic model consistent with reported data. Moderate GSK-3β activity levels are optimal to maintain microtubule bundles: Unphosphorylated Tau binds the microtubule shaft and prevents Eb1 from binding the lattice. Low levels of Shot phosphorylation ensure that Shot can interact with Eb1 and guide polymerizing microtubules (MTs) into parallel bundles. Low activity of GSK-3β leads to a strong association of Shot with the microtubule shaft and reduction of Eb1 binding, thereby losing its ability to guide polymerizing microtubules. Consequently, microtubules are disorganized. Hyperactivity of GSK-3β causes a loss of Tau and increase of Eb1 along the microtubule shaft. Eb1 comet size is reduced, and Shot is not able to efficiently bind plus ends and guide polymerizing microtubules. Schematic in (*A* and *D*) generated with Biorender.

We finally asked whether microtubule unbundling affects neuronal function. To test this, we measured larval crawling speed as a simple locomotor behavioral readout (41) (Fig. 6*A*). Third instar wandering larvae expressing hyperactive (*sggCA*) or inactive dGSK-3β (*sggDN*) crawled significantly shorter distances in 60 s (Fig. 6*B*).

Above we show that unbundling caused by hyperactive dGSK-3β was rescued by coexpression of Eb1, but not EGC-GFP. Similarly, crawling distance was restored only when Eb1-GFP was coexpressed (Fig. 6*B*). Inhibition of dGSK-3β induced microtubule curling via Shot. Phospho-deficient, but not phosphomimetic Shot variants triggered microtubule unbundling (Fig. 5*D*) and reduced crawling speed (Fig. 6*C*).

Together, these results show that misregulation of GSK-3β impairs neuronal function, with microtubule unbundling contributing directly to locomotor defects.

## Discussion

### A Specific Role of GSK-3β in Maintaining Axonal Microtubule Bundles.

We describe an evolutionary conserved mechanistic model of how GSK-3β maintains parallel microtubule bundles in neurons. We found that GSK-3β activity must be tightly balanced to maintain parallel bundles of microtubules; hyper- or inactivation of GSK-3β leads to curling of microtubules in fly and rat neurons. GSK-3β regulates microtubule bundling through the microtubule binding proteins Tau and Shot (Fig. 6*D*), two microtubule regulators that maintain microtubule bundling through Shot/Eb1-mediated guidance of microtubule polymerization (18). Shot guides polymerizing microtubules into parallel bundles by crosslinking actin and Eb1 (16). The Shot/Eb1 guidance mechanism breaks in different ways when GSK-3β is inhibited or hyperactive. In both situations, Shot-mediated guidance of polymerizing microtubules is reduced, leading to aberrant growth of unbundled microtubules toward the plasma membrane (18). GSK-3β hyperactivity causes a loss of Tau from microtubules, either through detachment and/or reduced protein abundance but not through changes on total *tau* transcript level. Whether or not individual Tau isoforms (RE, RL) partially compensate for this loss of major Tau isoforms remains to be verified but might explain the small difference between the tauGFP^w^ and tauGFP^M^ results. In any case, loss of *tau* results in Eb1 binding to microtubule shafts. Sequestration of Eb1 to microtubule shafts reduces Eb1 that is available to guide polymerizing microtubules into properly formed bundles by reducing Shot/Eb1 interaction. GSK-3β inhibition reduces microtubule guidance by Shot by reducing the affinity of Shot for Eb1.

Our data support a model where phosphorylation by GSK-3β balances two of Shot’s key functions – binding/stabilizing microtubules directly and guiding microtubule polymerization by binding Eb1 (16). Wu et al. (42) found that microtubule binding of the Spectraplakin ACF7 is regulated by GSK-3β, where ACF7 detaches from microtubules upon phosphorylation. We similarly observed that *Drosophila* Shot–microtubule association was reduced with phosphomimetic Shot. In addition, we found that Shot’s interaction with Eb1 depends on the phosphorylation state of Shot, where phosphomimetic Shot can still bind Eb1 despite being detached from microtubules. This switch could be the mechanism by which signaling coordinates and controls two key functions of Shot: 1) stabilizing and protecting microtubules when they are unphosphorylated and 2) guiding polymerizing microtubules into parallel bundles. This is reminiscent of a similar mechanism that was described for Clasp which contains two GSK-3β motives (43, 44). While Wu et al. did not directly analyze ACF7’s interaction with Eb proteins, they observed that directionality of microtubule growth was largely random in ACF7 KO cells. Expression of WT ACF7, but not phosphodeficient ACF7(S:A) rescued properly directed microtubule growth. This suggests that the role of GSK-3β in spectraplakin/Eb interaction and guidance of microtubule polymerization is evolutionary conserved. It is not yet clear how phosphorylation mediates a shift between the microtubule- and Eb1-bound states of Shot. Our structural in silico analysis using ColabFold/AlphaFold2 suggests that a key GSK-3β target cluster is in a linker region between Shot’s two Eb1 dimer-binding SxIP sites and the SxLP site (*SI Appendix*, Fig. S3*C*). Increasing the negative charge in this linker region may lead to a loss of microtubule affinity and strengthening interactions with Eb1, e.g. through binding of the SxLP site with an additional Eb1 dimer.

### Relevance of Microtubule Organization in Neurons.

Microtubules provide essential structural support for axons and are the substrate for all long-range vesicle transport. Unsurprisingly, proper maintenance of microtubule bundles is required for neuronal development and health. Defects in microtubule bundles can trigger axonal decay. Conditions where microtubules unbundle in culture conditions lead to microtubule curling and axonal swellings in brains which can impair vesicle transport and action potential propagation, key features of axonopathies (4, 18, 45). Pathological axon swellings in which microtubules bundles have disintegrated into loops or waves have been observed in aging, after injury, and in animal models of axonopathies (3). GSK-3β is the key coordinator of the parallel microtubule architecture in axons. Therefore, unbundling of axonal microtubules is a likely mechanism by which impaired GSK-3β activity leads to neuronal decay.

In our system, GSK-3β modulation affected neuronal Tau and Eb1 functions and we wondered if this had physiological consequences. There are several phenotypic changes that altered Tau activity/expression has been associated with in *Drosophila*: changes in habituation behavior and long-term memory through defects in mushroom body neuroplasticity (46), changed Ethanol sensitivity (47), altered resistance to neuronal oxidative stress through dysregulation of lipid droplet formation in glia cells (e.g., in retinas) accompanied by altered sleep behavior, reduced motor activity, and reduced survival (48) among others. Similarly, Eb1 dysfunction causes neuromuscular defects and defects in mechanosensory chordotonal organs in *Drosophila* (49). Therefore, we investigated if GSK-3β activity changes had functional consequences on crawling behavior in larvae. Indeed, dysregulation of dGSK-3β/sgg activity affected larval crawling. We show that interventions that restore microtubule bundling in primary neurons also improve larval crawling, indicating that the microtubule-unbundling phenotypes observed in culture have clear functional relevance in vivo.

Extensive prior work supports a central role for GSK-3β/Shaggy in neuronal development and maintenance. Embryos lacking *shaggy* show severe developmental defects (23). In differentiated neurons, dGSK-3β/sgg regulates axonal cargo transport (50) and constrains synaptic growth, with loss of regulation leading to structural and functional synaptic abnormalities (51) (52). We recently showed that hyperactive or inactive dGSK-3β causes visual defects (53) which might link back to the phenotypes described for Tau dysfunction in fly retinas (48). Together, these findings highlight the broad importance of GSK-3β in neuronal function. Future work should explore which specific roles and substrates of GSK-3β underlie the diverse phenotypes observed across neuronal contexts.

Our findings are important for neurodegenerative diseases in which GSK-3β is hyperactive and leads to hyperphosphorylation of Tau and the formation of neurofibrillary tangles, such as Alzheimer’s and Parkinson’s Disease. Much emphasis has been put on those aggregates. Work in flies and mouse models suggested that loss of Tau alone does not cause severe phenotypes (35, 54) apart from mild behavioral and motor deficits [e.g., (55, 56)]. However, we suggest that loss of endogenous Tau function may have another important effect. GSK-3β hyperactivity detaches Shot in addition to Tau from microtubules, disrupting their redundant stabilizing roles. This combined loss has been shown previously to cause severe microtubule instability, impaired transport, and synaptic defects (57). We propose this as a mechanism by which hyperactive GSK-3β drives neuronal decay in neurodegenerative disease.

The loss of microtubule organization caused by GSK-3β dysregulation might not only be relevant in neurodegenerative diseases but in aging, where many neuronal axons are lost (58). A recent study found that Tau, Shot, and EB1 are essential for maintaining microtubules in aging axons. Loss of these proteins in aging neurons leads to the decay of microtubule bundles, which subsequently cause a decline in axons and synaptic terminals (45). Notably, GSK-3β expression increases with age (59, 60). Therefore, misregulation of GSK-3β activity could be an underlying cause for the loss of microtubule regulators and therefore microtubule integrity as we get older.

GSK-3β is a highly promising therapeutic target for various neurological disorders, from Alzheimer’s, Parkinson’s, and Huntington’s disease to neurodevelopmental and mood disorder. However, trials involving global GSK-3β inhibitors have largely failed. We propose a model that explains why global inhibition of GSK-3β has not been successful. Normal development depends on fine-tuned, spatiotemporal regulation of GSK-3β, which remains poorly understood. Deviating even slightly from optimal levels of GSK-3β leads to problems with neuronal development, function, and morphology.

## Materials and Methods

### Fly Stocks.

Lines for targeted gene expression were *UAS-sgg.S9A* [*UAS-sggCA*; constitutively active (CA) form of Sgg, (61)]*, UAS-sgg.A81T* [*UAS-sgg.DN;* inactive form of Sgg, (20)], *UAS-Eb1-GFP and UAS-shot-Ctail-GFP* (*EGC-GFP*) (16)*, UAS-GFP* (generated by us). Loss-of-function mutant stocks used in this study were *sgg^1^* [hypermorphic allele, (62)], *dtau^KO^* (hypomorphic allele; (35), *tau^CRIMIC^*[CR70012; (63)], *tau^Def^* [Df(3R)BSC49], *shot^3^* [the strongest available allele of *short stop*; (64, 65)]. Gal4 driver lines used were the pan-neuronal lines *elav-Gal4* (1^st^ and 3^rd^ chromosomal, both expressing at all stages) (66). Protein trap line used was P{Wee}tau[304] [tauGFP; (33, 67)] and Mi{MIC}tau^MI03440^ (34). To induce gene expression with RU486, UAS constructs were expressed using an *elavGal4-GeneSwitch* driver line [RRID:BDSC_43642, (68, 69)]. Gene expression was induced by adding 200 mg/mL RU486 to cell culture media. Oregon R stocks, heterozygous crosses of UAS, or Gal4 lines with Oregon R or uninduced fly lines were used as controls as indicated.

### Generation of Shot-eGFP CRISPR/Cas9 Lines.

The five C-terminal exons of Shot (shot-RH, FBtr0087621), coding for the C-tail and SxIP sites, were excised and replaced with fused versions, including eGFP or phospho-deficient/mimetic variants. This resulted in ShotWT-eGFP (WT with an unmodified GSK-3β target site), phospho-deficient Shot (Serines mutated to Alanines), and phosphomimetic Shot (Serines mutated to Aspartic Acid). The Shot-eGFP sequences were synthesized as gBlocks (IDT). CRISPR gRNAs (5′ gRNA: GGAGGCTCTCGTGCCGGCTC, 3′ gRNA: GCTATAGGAAGCCACCGTTA) were identified using the CRISPR optimal target finder and cloned into pCFD5 via Gibson assembly. CRISPR donor plasmids with 0.8 kb 5′ and 3′ homology arms, flanking the Shot-eGFP sequences, were assembled in pUC57. Constructs were midi-prepped, injected into y[1] sc[1] v[1];; {y[+t7.7] v[+t1.8]=nanos-Cas9}attp2 flies, and eGFP-positive candidates were confirmed by Sanger sequencing. For more details, see supplementary methods.

### *Drosophila* Primary Cell Culture.

*Drosophila* primary neuron cultures were prepared as described (17, 18, 70). Stage 11 embryos were bleached (90 s), sterilized in 70% ethanol (<5 min), washed in Schneider’s medium with 20% FCS, and homogenized in 100 μL dispersion medium (0.005% phenylthiourea, 1% Pen/Strep in HBSS) for 4 min at 37 °C. Dispersion was stopped with 200 μL Schneider’s/FCS, cells pelleted (4 min, 650 g), and resuspended in 30 μL Schneider’s/FCS per coverslip. Typically, three coverslips per condition (6 to 8 embryos each) were cultured. Drops were placed in culture chambers, covered with concanavalin A–coated coverslips (5 μg/mL), allowed to adhere for 90 to 120 min, then inverted and grown as hanging drops at 26 °C.

### Rat Hippocampal Primary Cell Culture.

All experiments involving vertebrate animals were approved by the Institutional Animal Care and Use Committee at RPI.

Primary hippocampal neurons were cultured as previously described (71). In short, E18 rat hippocampi were dissected, trypsinized, dissociated, and plated on 18 mm glass coverslips coated with poly-l-lysine. Cultured neurons were grown in N2-supplemented Minimal Essential Medium at 37 °C with 5% CO_2_. Stage 4/5 hippocampal neurons (5 to 11 DIV, respectively) were transfected with Lipofectamine 2,000 (Thermo Fisher, Cat# 11668019) following the manufacturer’s instructions. To image stage three neurons, dissociated neurons were electroporated by nucleofection (AMAXA) using standard protocols prior to plating. Cells were allowed to express for 24 h before imaging or fixation.

### Immunohistochemistry.

Primary neurons were fixed for 30 min at room temperature (4% paraformaldehyde (PFA) in 0.05 M phosphate buffer, pH 7 to 7.2). For anti-Eb1 staining, cells were fixed with −80 °C-cold +TIP fix (90% methanol, 3% formaldehyde, 5 mM sodium carbonate, pH 9) for 10 mins at −20 °C and then washed in PBT (PBS with 0.3% TritonX). The following staining reagents were used: anti-tubulin (clone DM1A, mouse, 1:1,000, Sigma); anti-DmEb1 (gift from H. Ohkura; rabbit, 1:2,000) (49); anti-GFP (rab, 1:500, ab290, Abcam); anti-HA (rat, 1:200, 3F10, Sigma-Aldrich); F-actin was stained with Phalloidin conjugated with TRITC/Alexa647, FITC or Atto647N (1:100; Invitrogen and Sigma). Specimens were embedded in ProLong Gold Antifade Mountant (ThermoFisher Scientific).

### Microscopy and Data Analysis.

For standard imaging, we used AxioCam 506 monochrome (Carl Zeiss Ltd.) or MatrixVision mvBlueFox3-M2 2124G digital cameras mounted on BX50WI or BX51 Olympus compound fluorescence microscopes. For the analysis of primary neurons, we used the following parameters:

Microtubule curling in axons was quantified as the microtubule disorganization index [MDI; (17, 18)], calculated by dividing the total area of disorganized microtubules by axon length. Axon length was traced using ImageJ’s segmented line tool, and disorganized regions were outlined with the freehand selection tool. Analysis was automated using Fiji/ImageJ macros: axondisorg_table or axon-disorganization-from-rois (https://github.com/avmann).

The amount of Eb1 at microtubule comets was approximated from Eb1 staining on microtubule ends by multiplying comet mean intensity with comet length [described here (18)].

### In Vitro Kinase Assays.

For recombinant protein expression and purification, see supporting methods. Constitutively active GSK-3β protein was obtained from MRC PPU (Dundee), rabbit monoclonal thiophosphate ester antibody (clone 51-8) and p-Nitrobenzyl mesylate (PNBM) from Abcam (Cambridge, UK) and ATPγS from Biorbyt (Cambridge, UK). All other reagents were obtained from Sigma (Dorset, UK).

Thiophosphorylation assays were conducted in 25 µL reactions at 30 °C for 3 h, containing 400 ng GSK-3β, 3 µg substrate (GST or GST-peptides), and reaction buffer (50 mM HEPES pH 7.5, 10 mM MgCl2, 100 mM NaCl, 1 mM DTT). ATPγS (1 mM) was added to initiate the reaction. After 3 h, 2.5 mM PNBM was added, and reactions were incubated at room temperature for 2 h. Reactions were quenched with 4X Laemmli buffer, separated on 12.5% SDS-PAGE, transferred to PVDF, and analyzed for thiophosphorylation by Western blot using anti-thiophosphate ester (1:10,000) and anti-rabbit-HRP (1:5,000) with ECL detection. Coomassie-stained gels confirmed equal substrate loading. Densitometry was performed using ImageJ.

## Supplementary Material

Appendix 01 (PDF)

Dataset S01 (XLSX)

Dataset S02 (XLSX)

Dataset S03 (XLSX)

Dataset S04 (XLSX)

Dataset S05 (XLSX)

Dataset S06 (XLSX)

Dataset S07 (XLSX)

Dataset S08 (XLSX)

## Data Availability

All quantitative data and representative images are included in the article and/or supporting information and datasets.
