# Supplementary material for: GSK-3β coordinates axonal microtubule organization through Shot and Tau
Source: Proc Natl Acad Sci U S A. 2026 Feb 17;123(8):e2516746123. doi: 10.1073/pnas.2516746123 (PMC12933142; doi:10.1073/pnas.2516746123)
Supplement: Supplementary file 1 — Appendix 01 (PDF) [file pnas.2516746123.sapp.pdf]

PNAS

# Supporting Information for

## GSK-3β coordinates axonal microtubule organization through Shot and Tau

André Voelzmann[a,b], Lubna Nuhu-Soso[c], Alex E. Roof[c], Sanjai Patel[d], Hayley Bennett[e], Antony Adamson[e], Gareth J. O. Evans[c], Marvin Bentley[f] and Ines Hahn[b,c,1], ✉

a) School of Environmental and Life Sciences, Faculty of Science and Engineering, University of Hull, Hull, HU6 7RX, United Kingdom

b) Manchester Academic Health Science Centre, Faculty of Biology, Medicine and Health, School of Biological Sciences, Division of Molecular and Cellular Function, University of Manchester, Manchester, M13 9PL, United Kingdom

c) York Biomedical Research Institute and Department of Biology, University of York, York, YO10 5DD, United Kingdom

d) Manchester Fly Facility, Faculty of Biology, Medicine and Health, School of Biological Sciences, University of Manchester, Manchester, M13 9PL, United Kingdom

e) Genome Editing unit, Faculty of Biology, Medicine and Health, School of Biological Sciences, University of Manchester, Manchester, M13 9PL, United Kingdom

f) Department of Biological Sciences and the Center for Biotechnology and Interdisciplinary Studies, Rensselaer Polytechnic Institute, Troy, NY, 12180, USA

[1]To whom correspondence may be addressed. Email: ✉ ines.hahn@york.ac.uk.

**This PDF file includes:**

- Supporting Information Text
- Figures S1 to S4
- Tables S1 to S2
- SI References

**Other supporting materials for this manuscript include the following:**

Datasets: Figure 1 supporting data to Figure 6 supporting data, Figure S1 supporting data and Figure S3 supporting data.

**Supporting Information Text**

**Supporting methods**

Recombinant protein expression and purification

Primers were designed for MAP1B, shot and shot mutant peptide sequences with 5' EcoRI and 3' XhoI sites, annealed and ligated into pGEX-4T-1 (GE Healthcare). Peptide sequences are as follows; ERLSPAKSPSLSPSPPSPIEKT for MAP1B, SRAGSKPNSRPLSRQGSKPPSRHGS for shot and ARAGAKPNARPLARQGAKPPARHGA for shot mutant. Empty pGEX-4T-1 plasmid was used for GST only.

All constructs were expressed in BL21 E. coli at 37°C. Cultures at OD600 = 0.8 were induced with 1 mM Isopropyl β-D-1-thiogalactopyranoside (IPTG) for 3 h at 37°C with shaking at 200 rpm. Bacteria were harvested by centrifugation at 5000 x g for 15 min at 4°C and pellets were stored at -70°C overnight. Pellets were resuspended in 20 ml of PBS containing 1 mM phenylmethylsulfonyl fluoride (PMSF), 1X protease inhibitor cocktail and 6.65 mg of lysozyme. After incubation on ice for 30 min, 1 % (v/v) Triton X-100 and 5 mM DTT were added followed by 6 x sonication on ice for 30 s on and off for a total of 5 min 30 s at 17 kHz. The lysate was clarified by centrifugation at 12,000 rpm for 30 min at 4°C and the supernatant was incubated with 3x PBS pre-washed Glutathione Sepharose 4B resin for at least 1 h at 4°C with agitation. Glutathione resin was recovered by centrifugation, washed and protein was eluted in elution buffer (100 mM Tris pH 8.0, 20 mM glutathione, 100 mM NaCl). Purified protein was stored at -70°C until use.

RNA isolation

RNA isolations were performed for 5-6 independent repeats per genotype of the following genotypes:
*$w^{1118}$, $Shot^{WT}$-eGFP, $Shot^{SA}$-eGFP, $Shot^{SD}$-eGFP, elavGal4>UAS-GFP, elavGal4>UAS-sggCA, elavGal4>UAS-sggDN*

For each sample, wandering L3 larvae were washed in distilled water and their brains dissected out in 1x PBS and freed from discs and glands.

12 brains per sample were pooled in 200 uL StabiLyse buffer on ice, then supplemented with 200 uL RNAse-free water and ground using a pistil. 7.5 uL Proteinase K was added to the samples and incubated for 20 minutes at 55°C shaking. Afterwards, samples were spun down for 2 min at 16k x g and supernatant transferred to a new tube and an equal volume of StabiLyse buffer was added. RNA isolation then followed the NEB Monarch Spin RNA Isolation kit manual. RNAs were eluted in 30-40 uL RNAse-free water. RNAs were sampled and concentrations were determined by NanoDrop measurements. RNAs frozen at -80C for long-term storage.

cDNA synthesis

cDNA and -RT controls were prepared from 300-350 ng RNA (250-300 ug for +RT, 50 ug for -RT) using the Qiagen Quantitect Reverse Transcription kit following manufacturer's instructions.

Realtime qRT-PCR

Realtime qRT-PCRs were run in 384 well plates on a ThermoFisher QuantStudio 7 Pro Realtime PCR system. Each 10 uL reaction contained 4.6 - 5 ng cDNA, 800nM concentration of forward and reverse primers and 1x ThermoFisher PowerUP SYBR in a total reaction volume of 10 uL. qPCRs were run for 40 cycles following manufacturer's instructions and were followed by a melt-curve analysis. Primer efficiencies were determined experimentally via dilutions series of cDNA (3 additional dilution steps at 1:5 dilution). Reactions were performed in duplicates for 5-6 biological repeats per genotype. For primers used see Table S2.

Gene expression analysis

To determine relative expression levels, gene expression of target genes was normalised to the average CT value of mEFTu1, RpL32, and eIF1a as reference genes. Relative expression was determined using the delta-delta CT method while including primer efficiencies.
$relative\ gene\ expression = \frac{(E_{GOI})^{\Delta Ct\ GOI}}{GeoMean[(E_{REF})^{\Delta Ct\ REF}]}$. Each relative gene expression per genotype

sample was then normalised to the average expression of the respective control conditions. Three sample sets in which the average reference gene expression differed by more than 3 cycles or gene expression could not be detected were discarded before analysis. Differences in gene expression were analysed using a non-parametric Kruskal–Wallis one-way ANOVA with post hoc Dunn's test against the respective control conditions.

*Drosophila* Behaviour Assay - Larval Crawling

Wandering third instar larvae were then kept on this dish for up to an hour in a room warmed to 25$^{o}$C and selected individually for crawling. The lid of a six-well polystyrene plate coated in 1% agar and warmed to 25$^{o}$C was used for the crawling assay. Individual larvae were gently transferred with a paintbrush to the coated lid and put into a Zantiks behaviour system (Zantiks MWP Z2 system, maintained at 25$^{o}$C) and crawling recorded for 60s after 10s habituation period. The code to collect larvae crawling data was written in Zanscript coding language and retrieved distance crawled in mm at 30s and 60s (below). All equipment was pre-warmed to 25$^{o}$C. Data was analysed in GraphPad Prism v10, statistical significance revealed with t-test or non-parametric Mann-Whitney (depending on normality of distribution). An average of 80 larvae were collected for each condition across minimum three separate days. No larva was recorded twice and those that burrowed into the agar during the 60s crawling recording were disregarded.

**Zantiks Drosophila Crawling Code - Zanscript**

Zanscript – drosophila_larval_crawling Script 2 measurements 30s and 60s of distance – to get total distance at 60s do sum of both

```
# Zanscript on-line development - enter script below.
#aPD
# define experiment requirements
DEFINE TIME_BIN 30
DEFINE NUM_TIME_BINS 2
DEFINE NUM_SAMPLES 1
DEFINE VIDEO_LENGTH 70
# define the animal model tracking requirements (dependent on animal size)
SET(TARGET_SIZE,2)
SET(DETECTOR_THRESHOLD,6)

#define temperature
SET(THERMOSTAT,25)

# define auto reference tracking requirements
SET(AUTOREF_MODE,MOVEMENT)
SET(AUTOREF_TIMEOUT,10)

#defining the tracking and track length
DEFINE X_DRAWTRACKS 30011
DEFINE X_TRACKTIME  30016

ACTION MAIN

   LOAD(ARENAS,"aPD.bmp")

   SET(GPO6,1)
    SET(GPO7,1)
    SET(GPO8,1)
   VIDEO(VIDEO_LENGTH,"Zeb_LDT_1fps") #timelapse at 1 frame per sec

   LOGCREATE("TEXT:TIME|TEXT:TEMP|TEXT:CONDITION|TEXT:")
    LOGAPPEND("TEXT:A1")
```

```
    LOGRUN()

    AUTOREFERENCE()
    SET(X_DRAWTRACKS,1)
    SET(X_TRACKTIME,30)

    INVOKE(LOCOMOTION_TEST,NUM_TIME_BINS)

COMPLETE

ACTION LOCOMOTION_TEST
    SET(COUNTER1, COUNTER_INC)
    LOGDATA(DATA_SNAPSHOT, "BEGIN")
    WAIT(TIME_BIN)
    LOGDATA(DATA_SNAPSHOT, "END")
    LOGDATA(DATA_SELECT,"BEGIN")
    LOGDATA(DATA_DELTA,"END")

    PICTURE("photo_arena")
    LOGCREATE("RUNTIME|TEMPERATURE1")
    LOGAPPEND("COUNTER1|TEXT:DISTANCE|ARENA_DISTANCES:*")
    LOGRUN()
COMPLETE
```

*Colabfold/Alphafold2 prediction of EB1 and Shot-C-terminus multimer*

ColabFold/Alphafold2-multimer v1.5.3 was run via ChimeraX (1, 2). As Eb1 is a dimer, 2x Drosophila Eb1-PA and 1x Shot-PE C-terminal amino acids 4917-5201 were used as input. Colabfold was run in alphafold2_multimer_v3 mode with alignment and templating through MMseqs2 (3), HHsearch (4) and the PDB100 database (5, 6). The prediction used 5 seeds and 3 recycles. The best 3D model was visualised and domains highlighted in different colours using ChimeraX software.

**Supplementary Figures**

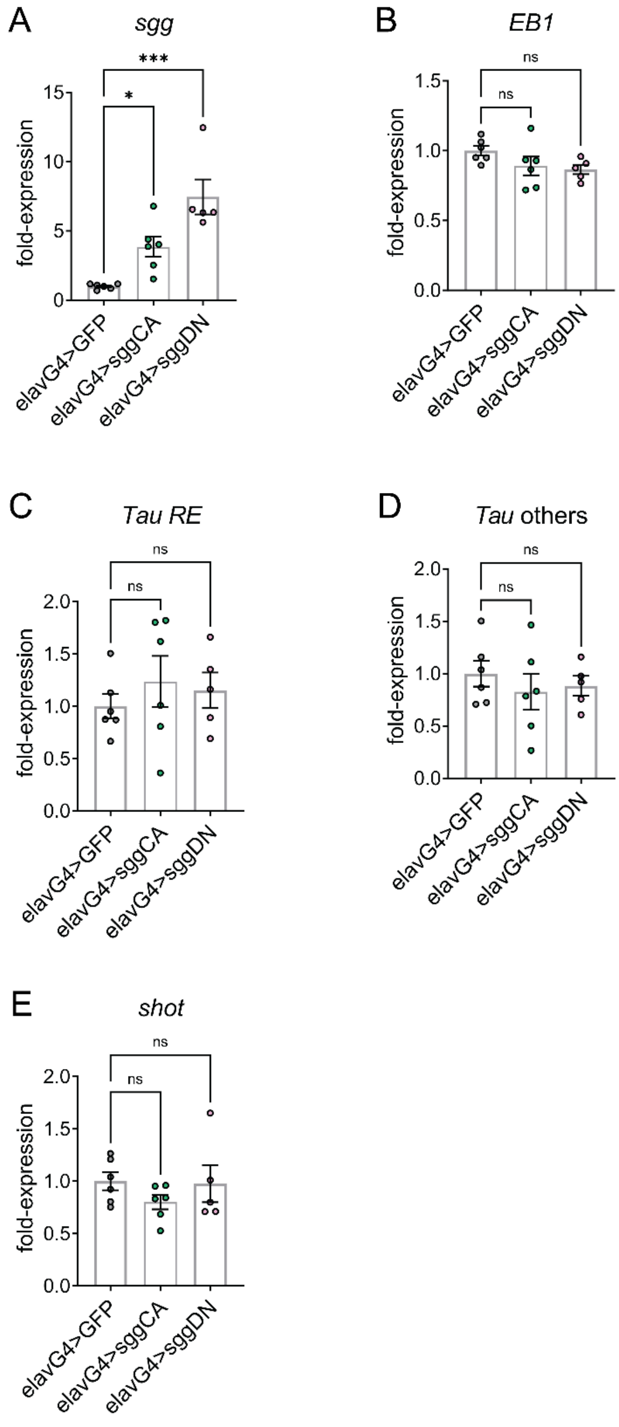


**Fig.S1 Expression of *sggCA* or *sggDN* does not affects expression of *Tau*, *shot* or *Eb1***

*UAS-sggCA* and *UAS-sggDN* were overexpressed using elav-Gal4 as driver line. *UAS-GFP* driven by *elav-Gal4* served as control. While brains dissected from L3 wandering stage larvae showed a robust and significant overexpression of *sgg* (A), the expression of *Eb1* (B), *Tau* (*TauRE* and all other isoforms, C,D) and *shot* (all isoforms, E) did not significantly change. Data are normalised to the average expression of *elav-Gal4* > *UAS-GFP*. Bar height indicates average

expression, whiskers SEM. Individual points represent biological repeats. For raw data see Dataset S7.

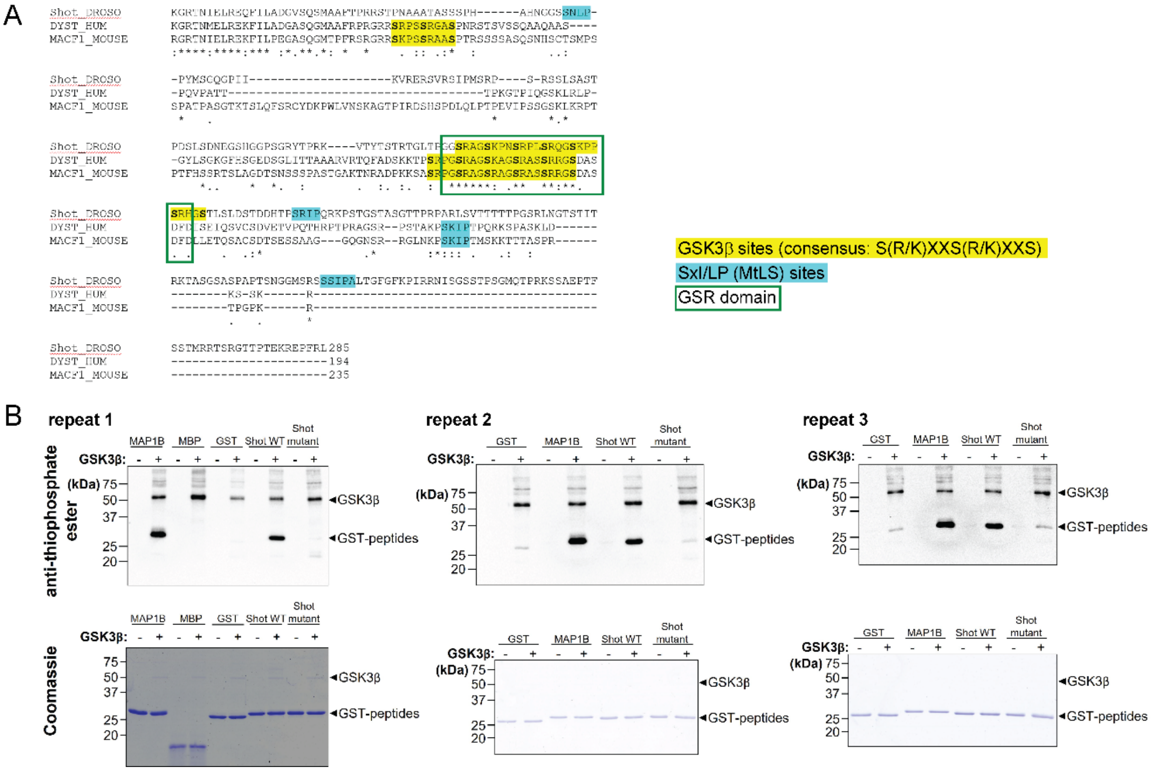


**Fig.S2 Phosphorylation of the C-terminus of Shot by GSK-3β**

**A)** Sequence alignment of the C-termini of *Drosophila* Shot (shot-RE FBpp0086744), human Dystonin (DST-001, ENST00000370788) and mouse MACF1 (Macf1-201, ENSMUST00000084301) highlighting the GSR domain (square), EB-binding SxIP sites (blue) and GSK-3β consensus site (yellow). **B)** Western blots of Coomassie gels of three repeats of the in vitro thiophosphorylation kinase assay Schematic representation of Shot; highlighting the verified ACF7 and putative Shot GSK-3β target site (consensus S/T S/T) in the C-terminal microtubule binding region. GST only (negative control), GSK-3β target site of MAP1B (positive control; ERLSPAKSPSLSPSPPSPIEKT (7)), Shot$^{WT}$ and Shot$^{S>A}$ (see Fig. 4D) with or without GSK-3β. Coomassie shows protein loading, blots show phosphorylated peptides are labelled with α-Thiophosphate ester. For raw data see S4 Data.

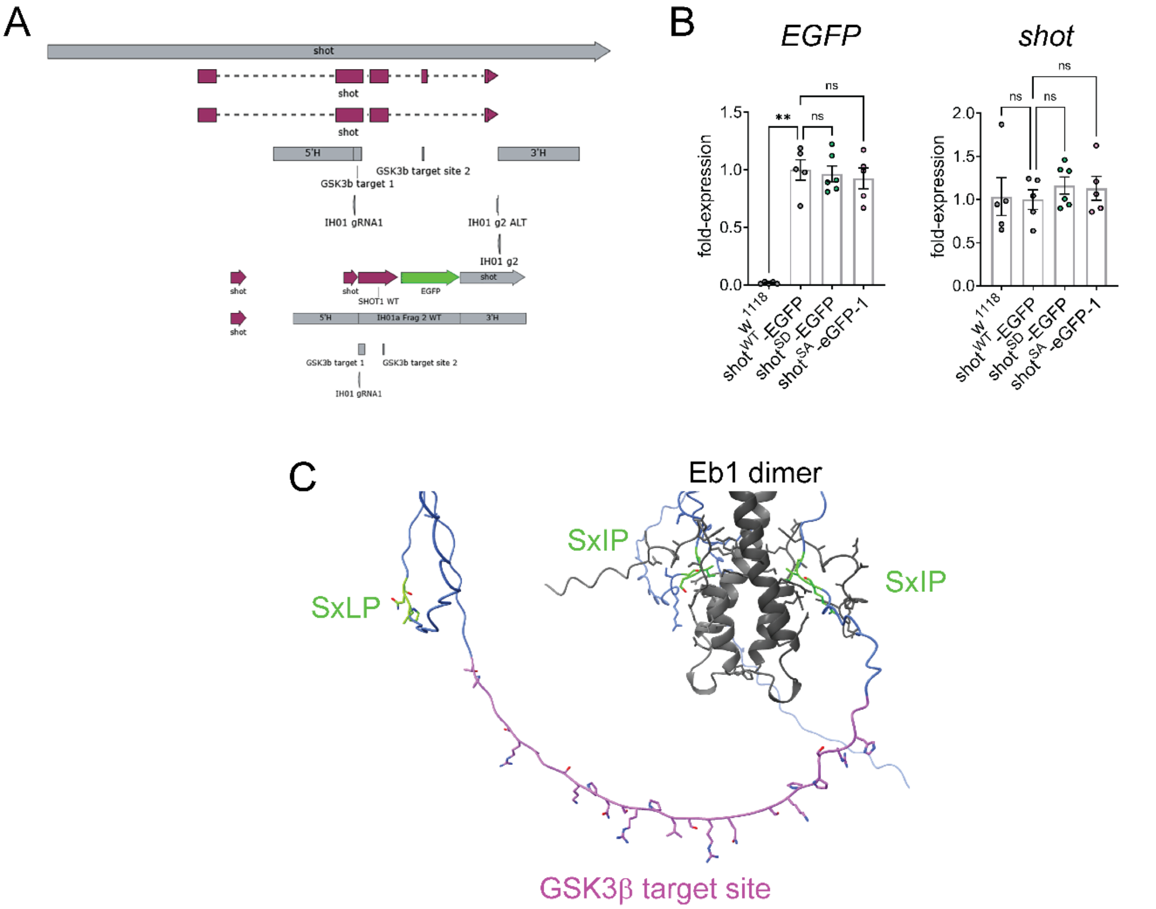


**Fig. S3 CRISPR/Cas9 mediated genomic targeting strategy to generate wildtype, phospho-mutant and -mimic *shot* alleles.**

**A)** The five C-terminal exons of *shot*, 5' and 3' homology arms and targeting sites for guide RNAs are depicted as well as an exemplar homologous repair construct ($Shot^{WT}$). **B)** New *shot* alleles in their endogenous locus were verified through real-time qRT-PCR. cDNA was obtained from L3 larval brains. *$Shot^{WT}$-eGFP*, *$shot^{SA}$-eGFP and $shot^{SD}$-eGFP* showed the same level of *eGFP* expression whereas *eGFP* is not detected in w1118 controls. There are no significant differences in the expression of *shot* or *Eb1* between w1118 and the new *shot* alleles. For raw data see Dataset S8. **C)** ColabFold-derived multimer model indicating potential binding modalities of c-terminal shot SxIP sites to Eb1 dimer and the positioning of the dGSK-3β target sites in between the two SxIP and the SxLP sites. SxIP and SxLP sites in green, dGSK-3β target sites in magenta, Eb1 in gray and dark gray respectively.

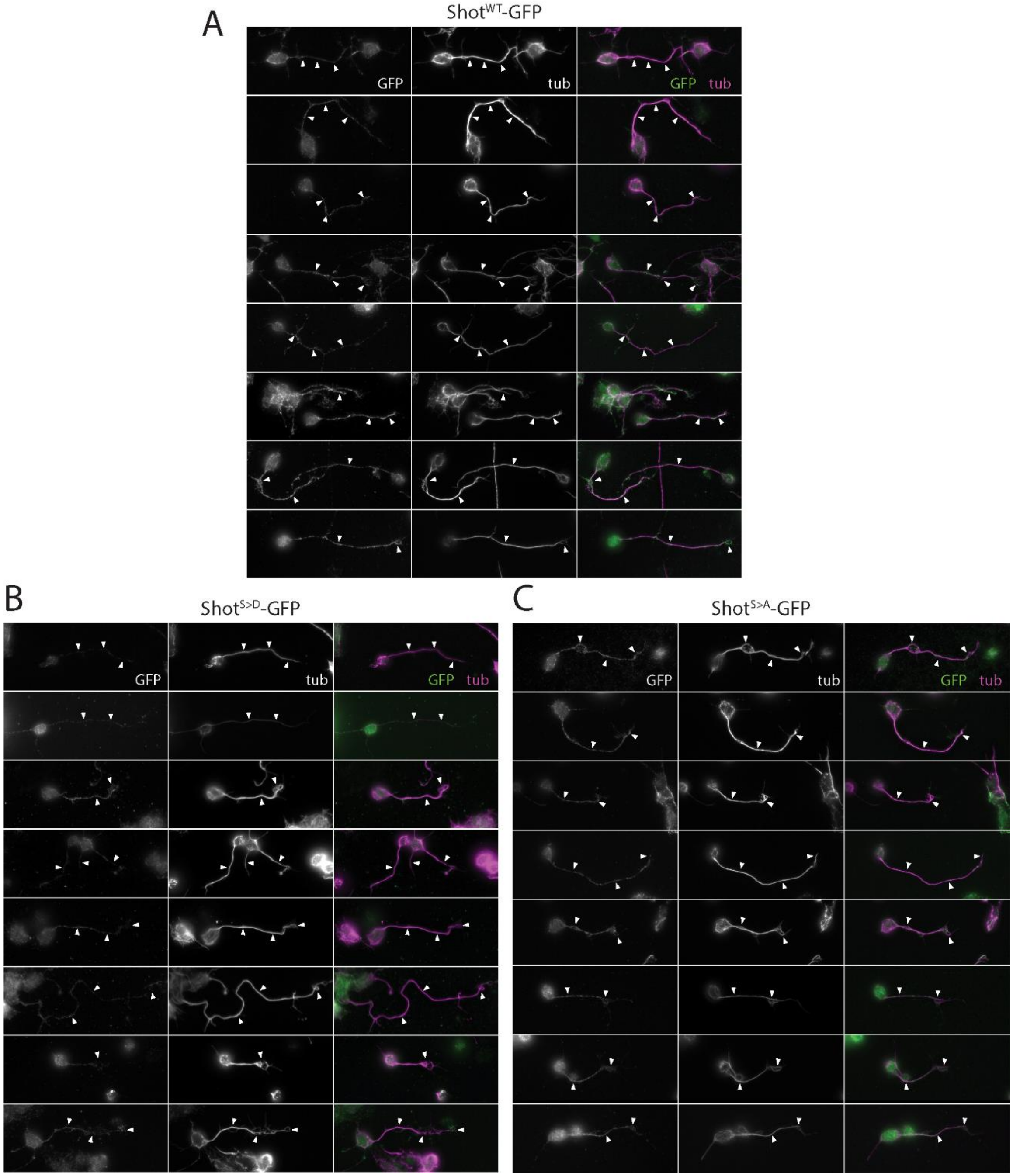


**Fig. S4 Phospho-deficient but not -mimetic Shot shows stronger, dotted localisation along microtubules – example images**

Representative images of *Drosophila* primary neurons cultured for 6HIV of *Shot*$^{WT}$ (A) *Shot*$^{S>D}$ (B) or *Shot*$^{S>A}$ (C) embryos from three independent cultures labelled for tubulin (magenta) and Shot (green, anti-GFP). Arrowheads point to microtubules.

**Tables**

**Table S1.** Primer sequences to generate transgenic *Shot*$^{WT}$*-GFP*, *Shot*$^{S>A}$*-GFP* and *Shot*$^{S>D}$*-GFP* lines

| Fragment | Template DNA | Primer(s) | Sequence |
|---|---|---|---|
| 1 (5'H) | Fly gDNA | Fragment 1 For | agtcacgacgttgtaaaacgacggccagtgaattctgctgctgcgccgc |
| | | Fragment 1 Rev WT | ggagccggcacgagatccgcctggcgtgagacccgt |
| | | Fragment 1 Rev Mimic | gtcgccggcacgatctccgcctggcgtgagacccgt |
| | | Fragment 1 Rev Mutant | ggcgccggcacgagctccgcctggcgtgagacccgt |
| 2 (cds-eGFP) | gBlocks | Fragment 2 For WT | ctcacgccaggcggatctcgtgccggctcca |
| | | Fragment 2 For Mimic | ctcacgccaggcggagatcgtgccggcgaca |
| | | Fragment 2 For Mutant | ctcacgccaggcggagctcgtgccggcg |
| | | Fragment 2 Rev | aacggtggcttcctattacttgtacagctcgtccatgcc |
| 3 (3'H) | Fly gDNA | Fragment 3 For | gagctgtacaagtaataggaagccaccgttatggct |
| | | Fragment 3 Rev | atctagatgcattcgcgaggtaccacaattttcactttcctattagtttaataacagaac |

**Table S2.** Primer sequences for realtime qRT-PCRs

| **Gene** | **Forward primer** | **Reverse primer** | **Annotated isoforms recognised** | **efficiency** |
|---|---|---|---|---|
| mEFTu1 | CATGTCCTTCATCCAACTGCA | AATGAGCTTGGTGTCTTCGCC | RA, RB (all) | 101.38 |
| RpL32 | GCTAAGCTGTCGCACAAATG | GTTCGATCCGTAACCGATGT | RA-RE (all) | 97.85 |
| eIF1a | CGGTCGTCTGGAGGCAAT | CCAATATGATGTCGCCCTGG | RA-RC (all) | 88.63 |
| eGFP | AACGTCTATATCATGGCCGA | GTGTTCTGCTGGTAGTGGTC | Not applicable | 101.02 |
| Eb1 | GAATAAACGCCTGTGCCA | GTTTATGCGCATATCCATCACC | RA, RB, RD-RG (all, RC not annotated) | 82.28 |
| Tau | CGAGGGCTAATCAGGATCAAAA | AGTCCTGGGCTAATGTCTGC | RE | 96.73 |
| Tau | CAAGAACCGCTTGCTCCT | GATCCGATCTTGGAGCGT | RA-RC, RF-RO (all but RE, RD not annotated) | 119.70 |

| | | | | |
|---|---|---|---|---|
| Shot | AAGCGACAGATTGAACAA CTC | GTTCCGTCAGTTCCAC AG | RA-RC, RE, RG-RQ, RX-RZ, RAA-RAD (all, RD, RF, RR not annotated) | 108.88 |
| sgg | CCGAATGTATCGTATATCT GCTCC | AGGACCTTGATGACCT CGAC | RA-RK, RM-RT (all, RL not annotated) | 103.88 |

**Datasets S1-S8 (separate files).**

Supplementary data sets with raw data for each Figure.

## SI References


1. M. Mirdita *et al.*, ColabFold: making protein folding accessible to all. *Nat Methods* **19**, 679-682 (2022).
2. E. C. Meng *et al.*, UCSF ChimeraX: Tools for structure building and analysis. *Protein Sci* **32**, e4792 (2023).
3. M. Mirdita, M. Steinegger, J. Sding, MMseqs2 desktop and local web server app for fast, interactive sequence searches. *Bioinformatics* **35**, 2856--2858 , pmid = 30615063 (2019).
4. M. Steinegger *et al.*, HH-suite3 for fast remote homology detection and deep protein annotation. *BMC Bioinform.* **20**, 473 , pmid = 31521110 (2019).
5. M. van Kempen *et al.*, Fast and accurate protein structure search with Foldseek. *Nature Biotechnology* 10.1038/s41587-023-01773-0 (2023).
6. H. Berman, K. Henrick, H. Nakamura (2003) Announcing the worldwide Protein Data Bank. pp 980 , pmid = 14634627.
7. N. Trivedi, P. Marsh, R. G. Goold, A. Wood-Kaczmar, P. R. Gordon-Weeks, Glycogen synthase kinase-3beta phosphorylation of MAP1B at Ser1260 and Thr1265 is spatially restricted to growing axons. *J Cell Sci* **118**, 993-1005 (2005).
